# Non-Coding RNA Editing in Cancer Pathogenesis

**DOI:** 10.3390/cancers12071845

**Published:** 2020-07-08

**Authors:** Giulia Romano, Michela Saviana, Patricia Le, Howard Li, Lavender Micalo, Giovanni Nigita, Mario Acunzo, Patrick Nana-Sinkam

**Affiliations:** 1 Department of Internal Medicine, Division of Pulmonary Diseases and Critical Care Medicine, Virginia Commonwealth University, Richmond, VA 23298, USA; giulia.romano@vcuhealth.org (G.R.); michela.saviana@vcuhealth.org (M.S.); patricia.le@vcuhealth.org (P.L.); howard.li@vcuhealth.org (H.L.); lavender.micalo@vcuhealth.org (L.M.); mario.acunzo@vcuhealth.org (M.A.); 2 Department of Molecular Medicine, University La Sapienza, 00161 Rome, Italy; 3 Department of Cancer Biology and Genetics, The Ohio State University, Columbus, OH 43210, USA; giovanni.nigita@osumc.edu

**Keywords:** non-coding RNA, editing, cancer

## Abstract

In the last two decades, RNA post-transcriptional modifications, including RNA editing, have been the subject of increasing interest among the scientific community. The efforts of the Human Genome Project combined with the development of new sequencing technologies and dedicated bioinformatic approaches created to detect and profile RNA transcripts have served to further our understanding of RNA editing. Investigators have determined that non-coding RNA (ncRNA) A-to-I editing is often deregulated in cancer. This discovery has led to an increased number of published studies in the field. However, the eventual clinical application for these findings remains a work in progress. In this review, we provide an overview of the ncRNA editing phenomenon in cancer. We discuss the bioinformatic strategies for RNA editing detection as well as the potential roles for ncRNA A to I editing in tumor immunity and as clinical biomarkers.

## 1. Introduction

In 1986, Benne and colleagues [1] first coined the term RNA editing to describe a frameshift observed in the highly conserved cytochrome oxidase II (coxII) gene present in the kinetoplasts of several trypanosomatid protozoans [1]. The investigators determined that genomic mitochondrial DNA (mtDNA) and its corresponding complementary DNA (cDNA) could be differentiated by the presence of additional thymidine residues in the cDNA sequence. Accordingly, they reasoned that this difference stems from the addition of uridine residues into the *coxII* mRNA transcript [1]. Following their observation, they identified similar editing events in related species [2].

In addition to these uridine-based modifications, other forms of RNA editing were being identified. In 1987, Powell and colleagues [3] and Chen and colleagues [4] both proposed that a cytidine-to-uridine (C-to-U) processing event was responsible for introducing a premature stop codon into the mammalian *apo-B100* mRNA transcript. In the following year, Bass and Weintraub [5], along with Wagner and colleagues [6], proposed that an adenine-to-inosine (A-to-I) shift accounted for the unwinding of dsRNA. As an extension to these initial findings, investigators sought to identify the enzymes that were responsible for C-to-U and A-to-I editing. Collective efforts from Teng and colleagues [7] and Navaratnam and colleagues [8] established that a cytidine deaminase, referred to as apolipoprotein B [apoB] messenger RNA [mRNA] editing catalytic polypeptide (APOBEC), was responsible for the C-to-U editing observed in *apo-B100*. Kim and colleagues [9] similarly proposed that double-stranded adenosine deaminases (DRADA) were responsible for widespread A-to-I editing events, including those that were observed in neural glutamate-gated ion channels [10]. This family of enzymes, now called adenosine deaminases acting on RNA (ADARs), consists of several members, most notably: ADAR1, ADAR2, and ADAR3 [9,11,12,13].

**Figure 1:**
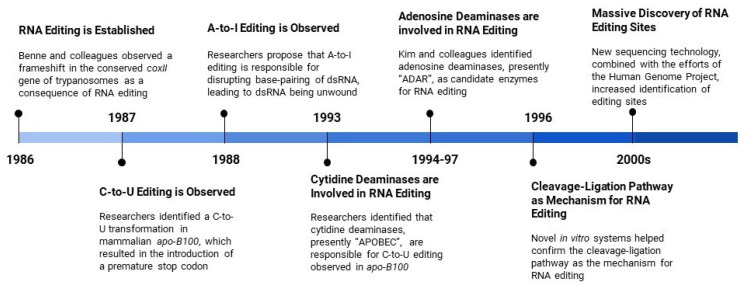
Milestones in RNA Editing Discovery.

Identifying the mechanisms responsible for these RNA editing events was initially complicated by the lack of a template RNA for comparison [14]. Consequently, when guide RNAs (gRNAs) were discovered by Blum and colleagues [15], researchers began generating theories for RNA editing. Prevailing theories suggested either a cleavage-ligation or transesterification mechanism [15,16], with the former being confirmed using in vitro systems of uridine-based modifications [17,18,19]. During this period, researchers attempted to identify specific editing sites, but discoveries were limited due to the technical complexities of available sequencing methods [20]. In response to the successes of the human genome project [21] and development of novel sequencing technologies [22], the identification of these editing sites increased exponentially, with the majority of A-to-I editing sites being discovered in *Alu* repeats [23]. Since these early contributions, the definition of RNA editing has expanded to encompass a host of post-transcriptional modifications, in which an RNA transcript is altered from its originating parent gene [14]. Moreover, RNA editing is now widely observed in a diversity of organisms in both coding and non-coding RNAs [24,25]. With this foundation established, researchers are shifting their focus towards examining the implications of these RNA editing events on biological functioning and disease progression [26,27,28,29]. In Figure 1, we summarize the milestones in RNA Editing Discovery.

## 2. RNA Editing Subtypes

Editing events involve either C-to-U or A-to-I base substitutions. Mechanistically, these events are derived from a hydrolytic deamination reaction: C-to-U substitutions are catalyzed by APOBEC, while A-to-I substitutions are catalyzed by ADAR [7,8,9,30]. Consequently, these deamination reactions cause an alteration in the original RNA sequence and disrupt the pre-existing nucleotide base pairing. As such, C-G bonds become U-A bonds, and A-U bonds become I(G)-C bonds.

### 2.1. Adenosine Deaminases Acting on RNA (ADARs)

The ADAR family of adenosine deaminases catalyzes A-to-I editing [9,31] (Box 1). In particular, this family consists of ADAR1, ADAR2, and ADAR3, which, in humans, are encoded in chromosomes 1, 21, and 10, respectively [32]. Structurally, the ADAR catalytic deaminase domain is localized in the C-terminal and its amino acid sequence is similar among the ADARs [33,34]. X-ray crystallography of ADAR2 reveals an active site that consists of four core amino acid residues involved in the coordination of a zinc ion: His394, Glu396, Cys451, and Cys516 [35]. Furthermore, an inositol hexakisphosphate (IP6) has been identified near the catalytic site, and it contributes to ADAR’s enzymatic activity and overall stability [35]. ADARs also contain up to three dsRNA binding domains (dsRBD) that directly interact with RNA [36,37]. One dsRBD (65 kDa) shows a αβββα secondary structure, which is highly conserved among the ADARs, and helps to facilitate contact with the RNA target [38,39].

#### 2.1.1. ADAR 1

ADAR1 consists of two isoforms. The interferon-inducible isoform ADAR1L (150 kDa) is involved in immune responses [40] and it is mainly expressed in the cytoplasm due to the presence of a nuclear export signal (NES) in its N-terminal domain [41,42]. The constitutive isoform ADAR1S (110 kDa) lacks a NES, so it is primarily localized in the nucleus [43,44]. Investigators have suggested that a shuffling system is responsible for the cytosolic expression of ADAR1S [45].

#### 2.1.2. ADAR2

ADAR2 is mainly localized in the nucleus and primarily expressed in brain tissue, where it has a fundamental role in neuronal development and activity [46]. It has been found expressed in other tissues, such as arterial vasculature, esophagus, and lung [31,47]. ADAR2 expression is regulated by the c-Jun transcription factor and by the transcriptional activator CREB [48,49] and it is predominately nuclear [32]. In 2015, Tommaselli and colleagues proposed the ADAR2 enzyme as a key factor for the editing balance of miRNAs involved in cancer [50].

#### 2.1.3. ADAR 3

ADAR3 is exclusively localized to the brain [13,51,52,53]. Although ADAR3 has a C-terminal region similar to the deaminase domain of ADAR2, its enzymatic activity has not yet been demonstrated [51,54]. The catalytic activity of ADAR1 and ADAR2 requires subunit homodimerization, while this event in vitro has not been reported for ADAR3 [55]. As such, the absence of ADAR3 homodimerization might explain its enzymatic inactivity [55]. Interestingly, ADAR3 expression negatively correlates with editing levels in the brain [56]. ADAR3 has been reported as a regulator of RNA editing in the brain by competing with ADAR2 for dsRNA substrates [57].

Box 1 **Adenosine Deaminases Acting on RNA (ADARs).**

ADAR1 is the INF-inducible isoform, ADAR1L (150 kDa), contains a NES sequence in the N-terminal region, and is mainly located in the cytoplasm. The constitutive isoform, ADAR1S (110 kDa), is localized in the nucleus.
ADAR2 is expressed primarily in the brain and is critical for neuronal development and activity.
ADAR3 is exclusively expressed in the brain. Its enzymatic activity has not been demonstrated thus far, but it may act as a regulator of RNA modification by competing with ADAR1 and ADAR2.
Structurally, the catalytic domain of ADAR is located in the C-terminal. In the well-studied ADAR2, the active site consists of four core amino acids: His394, Glu396, Cys451, and Cys516, which coordinate a zinc ion, and is stabilized by an inositol hexakisphosphate. Up to three dsRNA binding domains (dsRBD) are involved in RNA binding.
ADAR1 and ADAR2 catalytic activity requires homodimerization, but this event has not observed for ADAR3, probably explaining its enzymatic inactivity.

In 2015, Paz-Yaacov and colleagues determined that ADAR1 and ADAR2 were elevated in cancer as compared with normal tissues in a variety of tumors (invasive breast carcinoma, hepatocellular carcinoma, head and neck squamous cell carcinoma, bladder urothelial carcinoma lung adenocarcinoma, prostate adenocarcinoma, and thyroid carcinoma) [58]. This upregulation correlated with an increased number of edited sites detected and the Alu editing index (AEI). In the same year, Han and colleagues investigated the alteration in the number of editing events in tissues of several types of tumors as compared to the relative normal tissues. They reported an increase in A-to-I editing levels in lung adenocarcinoma, breast, bladder, head, and neck squamous cell, kidney renal cell carcinomas, and thyroid. Decreased editing events were found in kidney chromophobe and renal papillary carcinoma, while no difference was reported in liver, lung squamous, prostate, and stomach adenocarcinoma. The researchers also showed a positive correlation between the RNA editing and ADAR1 expression, but not ADAR2 and ADAR3, although a possible role of ADAR2 in editing regulation was not excluded [59].

### 2.2. Apolipoprotein B messenger RNA Editing Catalytic Polypeptide (APOBEC)

The human APOBEC protein family consists of 11 cytidine deaminases acting on nucleic acids and comprises both primary gene products and their alternatively spliced variants: activation-induced deaminase (AID), APOBEC1A (A1), APOBEC2A (A2), APOBEC3A (A3A), APOBEC3B (A3B), APOBEC3C (A3C), APOBEC3D (A3D), APOBEC3F (A3F), APOBEC3G (A3G), APOBEC3H (A3H), and APOBEC4 (A4) [60]. AID and A1 are encoded on chromosome 12; A2 and A4 are encoded on chromosomes 6 and 1, respectively, whereas all of the A3 are on chromosome 22 [61].

The cytidine deaminase catalytic domain is folded to form a structure containing five-stranded mixed β-sheets that are stabilized by six α-helices, where the overall sequence is α1-β1-β2-α2-β3-α3-β4-α4-β5-α5-α6 [62]. The reaction of C-to-U deamination requires a zinc ion that is coordinated by two conserved Cys and His residues; importantly, the maintenance of this zinc ion is necessary for the enzymatic activity of cytidine deaminases (reviewed in [62]).

Most of the APOBEC proteins contain one cytidine deaminase domain. However, A3B, A3D, A3G, and A3F comprise two domains in tandem, although only the C-terminal domain is catalytically active [62]. Thus, all of the APOBECs components have a zinc-coordinating deaminase domain (ZDD) motif (HxEx25-30PCx2-4C) within the cytidine deaminase fold [61]. Mechanistically, ZDD induce the hydrolytic removal of the exocyclic amine from cytidine or deoxycytidine with the consequential formation of uridine or deoxyuridine in single-strand DNA (ssDNA) or RNA [63].

AID activity is involved in immunoglobulin diversification [64,65] and its mutations have been found in patients with the autosomal recessive form of Hyper IgM syndrome [66]. Immunoglobulin diversification depends on genetic alteration, including gene conversion (GC), somatic hypermutation (SHM), and class-switch recombination (CSR), and AID seems to be involved in all of these events [65,66,67,68].

*A1* catalyzes the editing of C-to-U- in position 6666 and 6802 of Apolipoprotein B (ApoB) mRNA in the small mammal intestine and the liver of small species [60,69], although new targets have been identified [70]. Its activity requires the binding to a cofactor (A1CF), and a cis-acting nucleotide sequence (called mooring sequence) needed to recognize the editing site (reviewed in [71])

*A2* is expressed in skeletal muscles and cardiac tissue; A2 action is required for muscle development [72]. Interestingly, an increased expression of A2 has been linked to lung and liver tumorigenesis, and its constitutive expression induces accumulation of aberrant RNA sequence of PTEN and Eif4g2 genes in hepatocytes [73].

The main function of the A3 subfamily consists of the modification of endogenous and exogenous RNA and DNA (i.e., endogenous retroelements and exogenous viruses) [63]. Indeed, their antiviral activity against retroviruses and non-related viruses has been demonstrated [74,75,76]. Additionally, A3 overexpression has been found in several cancers, which suggests a possible function as a protooncogene [67].

When A4 is overexpressed in yeast and bacteria, this protein does not show any detectable cytidine deamination activity in DNA [77]. In fact, it weakly interacts with ssDNA [78]. A4 is mainly expressed in human testis, and it has been shown that it enhances the HIV-1 replication [78].

The APOBEC protein family plays an important role in cancer development and progression [60]. In 1995, Yamanaka and colleagues demonstrated, for the first time, that there exists a link between A1 and cancer by discovering that A1 transgenic mice and rabbits developed hepatocellular carcinoma [79]. Specifically, this finding appeared to be associated with hyper-editing and the subsequent downregulation of Novel A1 Target no.1 (NAT1; also known as eukaryotic translation initiation factor 4 gamma 2, or EIF4G2) [80]. Furthermore, A1 can bind and stabilize c-myc mRNA independently of its RNA-cytidine deaminase role [81]. Additionally, in a recent paper from Niavarani and colleagues, the researchers hypothesized a prognostic role for A1 in cancer [82]. Similarly, AID has also been associated with colorectal cancer [83], and it is aberrantly expressed in non-B cells, causing mutation(s) of non-IG genes that lead to an accumulation of p53 nucleotide alteration [84]. Recently, the important role of A3A and A3B in carcinogenesis has been well-established; specifically, their contribution to mutations in different cancers, such as bladder, cervical, breast, head and neck, and lung [85,86,87,88]. Chan and colleagues have also discovered that the A3A and A3B mutation signature is distinguishable in human cancer [89].

## 3. Editing of Non-Coding RNA in Cancer

Non-coding RNAs (ncRNAs) are untranslated RNA transcripts that are involved in the post-transcriptional and post-translational regulation of gene expression [24,25,90]. NcRNAs encompass: housekeeping RNAs, such as ribosomal RNA (rRNA) and transfer RNA (tRNA); small nuclear RNA (snRNA); small nucleolar RNA (snoRNA); and, regulatory RNAs. Regulatory RNAs can be further categorized by size into: small ncRNAs and long ncRNAs (lncRNAs). Small ncRNAs are less than 30 bp and they include: microRNA (miRNA), short interfering RNA (siRNA), piwi-interacting RNAs (piRNA), tRNA-derived fragments (tRFs), and tRNA halves (tiRNAs). lncRNAs are greater than 200 nucleotides [91,92,93,94]. With respect to post-transcriptional processing, A-to-I editing has been most studied in miRNAs and lncRNAs [32].

### 3.1. MicroRNAs (miRNAs)

During the last decade, an increasing number of investigators have analyzed the role and effect of A-to-I editing on microRNAs (miRNAs) [32,95]. MiRNAs are single-stranded RNA sequences that contribute to post-transcriptional regulation of gene expression [96]. They are transcribed into pri-miRNAs, which then undergo a series of cleavage reactions, catalyzed by Dicer and Drosha, to form a mature miRNA sequence [97]. RNA editing events that alter the pri-miRNA sequence have been shown to interfere with Dicer- and Drosha- mediated cleavage events, which in turn disrupts overall miRNA biogenesis [50,95,98,99]. It is estimated that 10–20% of miRNAs undergo editing at the pri-miRNA level. For example, approximately 20% of pri-miRNAs found in the brain are edited [95,100]. Furthermore, pri-miRNAs encoded by DNA viruses, such as Epstein–Barr and Kaposi, are found to be edited, as well [95]

RNA editing can also target mature miRNAs. To date, 2654 mature miRNA sequences have been identified in humans (miRbase 22.1, October 2018) [101]. Mature miRNAs are loaded into a multiprotein complex, called the RNA-induced silencing complex (RISC) [97,102]. The miRNA has a seed region, which helps to guide RISC to complementary mRNA sequences [97]. Mechanistically, miRNAs base-pair with target mRNAs, typically within the 3′ UTR, to negatively regulate their expression [97]. Therefore, when considering the miRNA’s *modus operandi*, editing of either the mRNA 3′UTR miRNA consensus site or the miRNA seed region can disrupt or induce miRNA silencing interactions [103,104,105]. Interestingly, it is rare to find new edited sites in mature miRNAs, which indicates that the expression of edited miRNA is relatively uncommon [95].

The altered expression of miRNA editing has been associated with cancer [106,107,108]. In general, miRNA editing is downregulated while the editing of the 3′ UTR of mRNAs is upregulated in cancers [106]. Interestingly, elevated miRNA editing levels are associated with longer survival [106]. As examples, ADAR1 can regulate miR-222 biogenesis at a transcriptional level and affect melanoma immunoresistance [109]. MiR-214 and miR-122 precursor and antisense editing mediated by ADAR2 is deregulated during hepatocarcinogenesis [110]. ADAR2 can reduce the expression of many oncomiRs and, in particular, ADAR2 can edit miR-222/221 and miR-21 precursors in vitro and in vivo with important effects on cell proliferation and migration [50].

To better distinguish cancer from non-cancer, investigators have proposed miRNA editing patterns as a classifier [107]. These miRNA editing profiles may also be leveraged for predicting specific cancer histologies [107]. For example, miR-589-3p contributes to cell migration and invasion, and is normally edited in brain tissue, but it is found to be downregulated in glioblastoma [111]. Similarly, the downregulation of miR-376a* editing was found to be correlated with an increase in cell invasiveness and a decrease in patient survival in glioblastoma [112]. In a recent paper by Nigita and colleagues, the investigators identified the deregulation of miRNA editing events between lung tumor and normal tissues [108]. In the same study and, for the first time, they observed a difference in the editing level of mature miRNAs in circulating exosomes [108]. Finally, another study demonstrated that ADAR2 enzyme downregulating DROSHA can decrease miR-15/16 expression increasing the oncogenic signal in lymphocytic leukemia [113].

#### Methods for MicroRNA Editing Detection and Functional Characterization

At the beginning of the 2000s, only a few RNA editing sites were detectable in miRNAs while using low-throughput methods [99,100,104,114,115]. With the advent of high-throughput sequencing (HTS) technologies, we have observed significant progress not only in terms of identification of novel genes, but also in the detection of post-transcriptional modifications, such as RNA editing. In fact, in the last decade, several groups have leveraged HTS technologies to profile and study the phenomenon of miRNA editing [32,116] moving from a few dozen to several hundred editing sites identified in miRNAs [117,118,119]. One of the first and most common HTS-based approaches for miRNA editing detection was utilized by Alon et al. [120,121], in which the authors identified 19 high-confidence A-to-I editing sites in mature miRNAs. Subsequently, Alon et al. implemented such a pipeline into a Web server [122], named DREAM (http://www.cs.tau.ac.il/~mirnaed/), allowing for users to upload their small-RNA sequencing data and profile miRNA editing. Recently, another group developed a more comprehensive HTS-based application [123], termed miRge2 (https://github.com/mhalushka/miRge), to concurrently profile edited miRNAs and miRNA isoforms, demonstrating that miRNA editing detection is strongly correlated with Alon’s pipeline.

**Figure 2:**
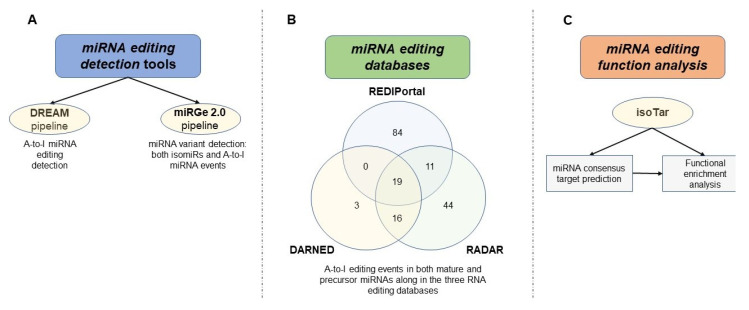
Methods for MicroRNA Editing Detection and Functional Characterization. (**A**) Two principal and most used pipeline (DREAM and miRGe 2.0) for miRNA editing detection. (**B**) Distribution of 177 putative and/or validated A-to-I RNA editing events in both mature and precursor miRNA molecules along with the three major RNA editing databases (DARNED, RADAR, and REDIPortal). (**C**) Illustration of isoTar, a web-based containerized tool designed consensus targeting prediction and functional enrichment analyses for miRNAs harboring editing sites and other.

As a result of the development of HTS technologies, hundreds of editing events in miRNAs have been compiled in an RNA editing database [118,124], called DARNED, (https://darned.ucc.ie/), and subsequently in an improved database that was created by Ramaswami and Li [119], named RADAR (http://rnaedit.com/). Recently, another resource was created by Picardi and colleagues, named REDIportal (http://srv00.recas.ba.infn.it/atlas/) [125], in which they have collected more than 4.5 million A-to-I events in 55 body sites of 150 healthy individuals. These databases contain editing events detected in both coding and non-coding genes and, in particular, there are a total of 177 putative and validated RNA editing sites in mature and precursor microRNA molecules (Figure 2).

Distefano et al. recently developed isoTar (https://ncrnaome.osumc.edu/isotar/), a Web-based application to perform a consensus miRNA targeting prediction and functional enrichment analyses for modified miRNAs, such as edited miRNAs and isomiRs [117], to study the biological effect of editing events. This tool is a containerized application that allows the user an easy installation in different Operative Systems, with no particular advanced technical skills.

### 3.2. Long Non-Coding RNA (lncRNA)

LncRNA are a heterogeneous group of ncRNAs that are longer than 200 nucleotides [126]. Though approximately 57K unique genes been classified as lncRNAs [127] (LNCipedia v.5), less than 200 of these sequences have been well characterized and studied [128]. LncRNA are involved in many cellular biology processes, including RNA splicing; regulation of gene, protein, and miRNA expression; and, harboring some hormone-like activities [129]. LncRNAs are also implicated in neurological [130] and cardiac disease [131], as well as in cancer [129,132].

There is evidence of a significant amount of A-to-I editing in lncRNAs, but the functional significance of this modification while under investigation is not fully understood [32]. Three possible roles for lncRNA editing have been proposed [133]. First, there is some evidence that highly-edited lncRNAs can be retained in the nucleus and be exported in the cytosol following cleavage of hyper-edited sites under stressful stimuli [134]. Second, once lncRNAs are edited, similar to miRNAs, they can be sensitized to degradation by Tudor-SN [98,133]. Third, similar to miRNAs, editing can modify lncRNA target sites [133].

Recently, a high throughput RNA-Seq based study demonstrated extensive RNA editing in the human transcriptome. The authors analyzed ∼767 million sequencing reads from poly(A)+, poly(A)−, and small RNA samples and they identified two lncRNAs that are edited at several sites Jpx (41 sites) and Malat1 (31 sites) [135].

lncRNAs may also interact with ADAR to regulate the expression of the target gene. Salameh and colleagues demonstrated that the prostate cancer antigen 3 lncRNA (PCA3) downregulated its target tumor suppressor gene PRUNE2 (a human homolog of the *Drosophila* prune gene) through an ADAR-mediated RNA editing mechanism. The authors also confirmed the co-regulation and RNA editing of PRUNE2 and PCA3 in human prostate cancer specimens [136].

In 2017, Luo and colleagues analyzed editing sites in coding transcripts and long non-coding transcripts of four samples from different cancer stages for the same patient [137]. They identified distinct editing profiles in different stages of cancer, and the impact on cancer-related pathways was increased with increasing malignant grade of samples. They hypothesized that the editing levels could have a functional impact during cancer development and progression [137].

### 3.3. tRNA

**Figure 3:**
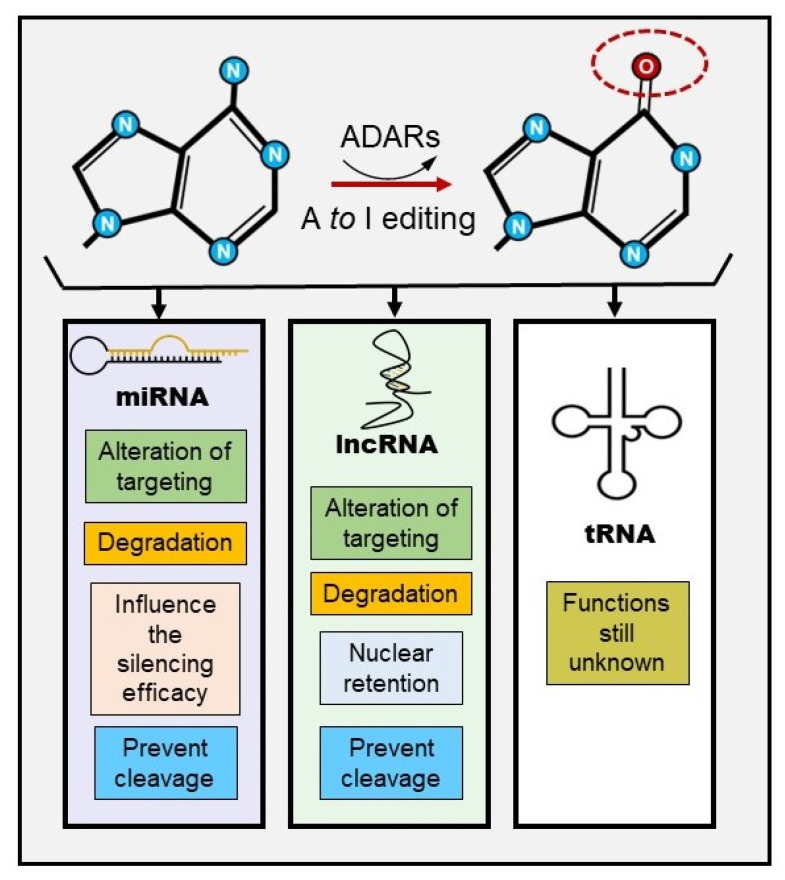
ncRNAs A-to-I biological consequences.

tRNAs are another class of ncRNA molecules that undergo A-to-I editing, but, in this case, the deamination is driven by a specific family of enzymes the adenosine deaminases acting on tRNA enzyme family (ADATs) [138]. These proteins are well conserved in metazoa and the sequence homology with ADARs suggests a model in which ADATs are ancestral to ADARs [53]. A-to-I editing in tRNA is well conserved and occurs in three specific positions 34, 37, and 57 of certain tRNA. Even though this phenomenon is well conserved, its role remains unknown. One hypothesis for editing in position 34 is that this modification can expand the numbers of recognized codons, but this hypothesis is the subject of debate [139]. In Figure 3, we summarize the downstream effects of ncRNA A-to-I editing.

## 4. The Role of RNA Editing in Tumor Immunity

Although aberrant patterns of RNA editing have been identified in a variety of cancers, the biological consequences of editing events on anti-tumor immune responses remain unclear. Asaoka and colleagues found that APOBEC3-mediated C-to-U RNA editing was common in tissues of The Cancer Genome Atlas (TCGA)-breast carcinoma (BRCA) cohort [140]. In TCGA-BRCA tissues, high levels of RNA editing were associated with enriched expression of immune-related gene sets from the Molecular Signatures Database Hallmark collection. By quantifying tumor-infiltrating immune cell subsets while using the CIBERSORT algorithm, the investigators showed that tumors with high levels of RNA editing had significantly more T cells, T cell subtypes (CD8+, CD4+, regulatory T cells, and γδ T cells), and M1 pro-inflammatory macrophages. TCGA-BRCA patients with high RNA editing in tumors had better disease-free and progression-free survival. These data suggest that APOBEC3-mediated RNA editing is associated with heightened immune activity and improved survival in breast cancer.

Zhang and colleagues designed a proteogenomics screening approach combining liquid chromatography-mass spectrometry and RNA sequencing data from primary human tissue samples and healthy tissue donor samples to identify RNA edited HLA ligands [141]. Focusing on the HLA-A ligands found for an ADAR1 editing site of cyclin I (*CCNI R75G*), the authors showed that edited CCNI peptides activated tumor-infiltrating lymphocytes from human melanoma tumors, suggesting edited CCNI peptides function as antigenic epitopes in vivo. Edited CCNI peptide-specific T cells also facilitated the killing of the HLA-A-expressing lymphoblast cell line T2. Thus, HLA-bound peptides derived from RNA editing can function as tumor antigens to elicit anti-tumor immune responses.

The editing of dsRNA by ADAR1 plays an important role in preventing aberrant and chronic innate immune response activation (reviewed in [142]). However, hyper-editing of RNA by elevated ADAR1 activity has been reported in some cancers [143,144,145,146,147]. Using a pooled in vivo genetic screening approach utilizing CRISPR-Cas9 genome editing in transplantable B16 melanoma tumors in mice treated with anti-PD-1 therapy, Manguso and colleagues identified ADAR1 as a top candidate to boost cancer immunotherapy [148]. To test whether the deletion of Adar1 sensitized tumors to anti-tumor immunity, Ishizuka and colleagues generated mouse B16 melanoma cells that lacked ADAR1 [149]. Adar1-null B16 tumors implanted in wild-type, immunocompetent animals were profoundly sensitized to anti-PD-1 antibody treatment. Adar1-null tumors had significantly increased proportions of T cells and natural killer cells; and, decreased proportions of myeloid-derived suppressor cells, M2 anti-inflammatory macrophages, and tumor-associated neutrophils. When compared with control tumor cells, Adar1-null cells showed a significant inhibition of viability and increased apoptosis when stimulated with IFNβ or IFNγ. The loss of MDA5 reversed the inflammation observed in Adar1-null tumors, which indicated that dsRNA sensing by MDA5 is required for the enhanced inflammation and immune infiltration in Adar1-null tumors. The deletion of β2 microglobulin (B2m) abolishes recognition of B16 melanoma cells by CD8+ T cells and renders them completely resistant to immunotherapy in vivo. The loss of ADAR1 restored sensitivity to immunotherapy and resulted in elimination of many of the B2m-null tumors, indicating that the deletion of Adar1 can overcome acquired resistance to immunotherapy.

These three studies indicate that RNA editing plays an important role in tumor immunity, but its precise role remains uncertain. Both Asaoka and colleagues and Zhang and colleagues show that RNA editing mediated by APOBEC3 and ADAR1 elicits anti-tumor immune responses in breast cancer and melanoma, respectively. In contrast, Ishizuka and colleagues demonstrate that the loss of ADAR1 in melanoma tumors increases tumor inflammation and overcomes resistance to immunotherapy. Thus, additional studies are required to understand the mechanisms, whereby RNA editing influences anti-tumor immune responses and whether RNA editing enzymes may represent novel targets for immuno-oncology therapy.

In contrast to general RNA editing, even less is known regarding the role of non-coding RNA editing in tumor immunity. ADAR1, in an editing-independent manner, has been shown to transcriptionally regulate the biogenesis of miR-222 and thereby Intercellular Adhesion Molecule 1 (ICAM1) expression, which consequently affects melanoma immune resistance [109]. However, to our knowledge, there are no published reports showing that miRNA editing regulates anti-tumor immune responses.

## 5. The Future of Noncoding RNA Editing as Biomarkers in Cancer

High throughput interrogation of the human transcriptome has led to the identification of post-transcriptional modifications that drive cancer biology. Transcriptional modifications, such as editing, have the potential to serve as markers of cancer stage, histology, prognosis and chemotherapeutic response. The dysregulation of A-to-I (e.g., ADAR) and C-to-U editors (AID/APOBEC) has been implicated in prognosis in carcinogenesis and both the innate and adaptive immune system [26,150]. As mentioned, Asaoka and colleagues demonstrated that APOBEC editing correlated with improved survival and enrichment of immune related gene expression in breast cancer [140]. Variants in RNA editing exist across several solid malignancies including colorectal cancer [151]. Permuth and colleagues identified that selected single nucleotide polymorphisms in ADAR correlated with susceptibility to epithelial ovarian cancers [152]. Investigators postulate that tumor ADAR1 expression may serve as classifier for therapeutic response and that cancer directed targeting of ADAR 1 may represent a viable approach to sensitization of poorly responsive tumors. For example, the deletion of ADAR1 resulted in the sensitization of several tumor types to immune checkpoint inhibition (ICI) [149].

**Figure 4:**
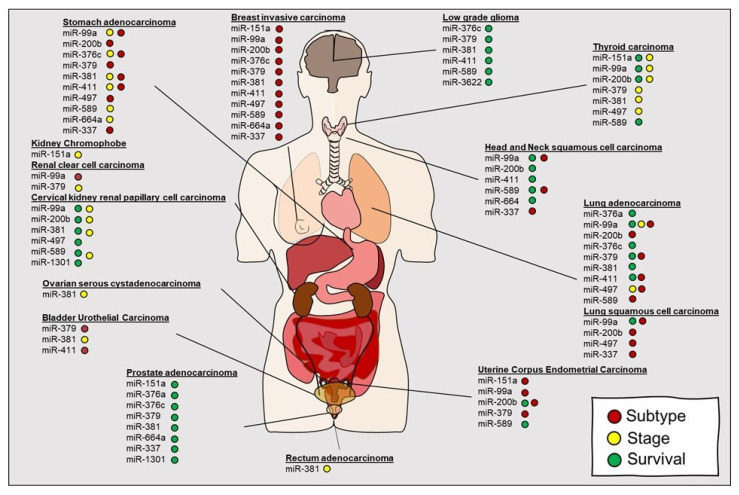
Schematic representation of miRNA editing hotspots in cancer correlated with key variables including survival, stage, and subtype. (Wang et al 2017).

The links between ADARs and AID/APOBEC editing proteins and miRNA are evolving. Both proteins can either alter absolute miRNA expression or indirectly impact miRNA targeting through editing 3′UTR sites. As we have discussed, one of the earliest observations by Choudhury and colleagues demonstrated that within high grade glioma of the brain, miR-376 was primarily in the unedited form correlating with tumor progression. Select A-to-I editing of miR-376 resulted in a shift towards a tumor suppressor like function [112]. This and subsequent studies started to suggest that editing within miRNA transcripts may occur across tumor types, including prostate cancer [153], thyroid cancer [154], lymphomas [155], and lung cancer [108,156]. Comprehensive analysis of miRNA editing regions by leveraging TCGA has facilitated the identification of cancer relevant regions for miRNA editing [107]. By the profiling of 8595 samples within 20 cancer types, the investigators identified 19 unique miRNA editing hotspots. Of note, the investigators identified both ubiquitous and cancer specific miRNA editing hotspots. They determined that editing within specific miRNAs, including miR-151a, miR-99a, miR-200b, miR-376c, miR-381, miR-411, and miR-664a correlated with key variables, including survival, stage, and subtype across some cancers suggesting translational application as biomarkers (Figure 4). Lastly, they conducted preliminary in vitro studies to suggest that editing in miR-200b as opposed to wild type miR-200b may redirect targeting of *ZEB1*, *ZEB2*, and *LIFR* and, thus, impact downstream signaling. Velazquez-Torres and colleagues demonstrated that selected editing of miR-378-3p resulted in a tumor suppressive phenotype in melanoma [157]. While many of these studies are encouraging, the suggested edited ncRNA classifiers still require more comprehensive testing, validation and independent evaluation for clinical utility.

## 6. Conclusions

While post transcriptional modifications, such as editing impact downstream biology of coding RNA in both malignant and benign human diseases, our understanding for editing in noncoding regions is just starting to emerge. With the advent of high throughput interrogation of the human genome, investigators have uncovered additional complexities of noncoding regions previously unrealized. This relatively new concept allows for us to implicate a new layer for both regulation and patterns of expression of such key genes as miRNAs and lncRNAs that extends beyond conventional approaches of examining absolute expression as biomarkers or linking unedited sequences to downstream targets. The functional mechanisms by which editing in noncoding regions may drive cancer initiation and progression are still in the early phases of investigation. However, it is increasingly evident that the deregulation of central mediators of editing, such as ADAR and APOBEC proteins across many cancers, suggests that editing contributes to many of the hallmarks of cancer. How such aberrations will translate to clinically inform decision making and their role as diagnostic and prognostic classifiers is unknown, but is sure to come to light in the near future.

## References

[1] Benne R., Van den Burg J., Brakenhoff J.P., Sloof P., Van Boom J.H., Tromp M.C. (1986). Major transcript of the frameshifted coxII gene from trypanosome mitochondria contains four nucleotides that are not encoded in the DNA. Cell.

[2] Maslov D.A., Avila H.A., Lake J.A., Simpson L. (1994). Evolution of RNA editing in kinetoplastid protozoa. Nature.

[3] Powell L.M., Wallis S.C., Pease R.J., Edwards Y.H., Knott T.J., Scott J. (1987). A novel form of tissue-specific RNA processing produces apolipoprotein-B48 in intestine. Cell.

[4] Chen S.H., Habib G., Yang C.Y., Gu Z.W., Lee B.R., Weng S.A., Silberman S.R., Cai S.J., Deslypere J.P., Rosseneu M. (1987). Apolipoprotein B-48 is the product of a messenger RNA with an organ-specific in-frame stop codon. Science.

[5] Bass B.L., Weintraub H. (1988). An unwinding activity that covalently modifies its double-stranded RNA substrate. Cell.

[6] Wagner R.W., Smith J.E., Cooperman B.S., Nishikura K. (1989). A double-stranded RNA unwinding activity introduces structural alterations by means of adenosine to inosine conversions in mammalian cells and Xenopus eggs. Proc. Natl. Acad. Sci. USA.

[7] Teng B., Burant C.F., Davidson N.O. (1993). Molecular cloning of an apolipoprotein B messenger RNA editing protein. Science.

[8] Navaratnam N., Morrison J.R., Bhattacharya S., Patel D., Funahashi T., Giannoni F., Teng B.B., Davidson N.O., Scott J. (1993). The p27 catalytic subunit of the apolipoprotein B mRNA editing enzyme is a cytidine deaminase. J. Biol. Chem..

[9] Kim U., Wang Y., Sanford T., Zeng Y., Nishikura K. (1994). Molecular cloning of cDNA for double-stranded RNA adenosine deaminase, a candidate enzyme for nuclear RNA editing. Proc. Natl. Acad. Sci. USA.

[10] Sommer B., Kohler M., Sprengel R., Seeburg P.H. (1991). RNA editing in brain controls a determinant of ion flow in glutamate-gated channels. Cell.

[11] Lai F., Chen C.X., Carter K.C., Nishikura K. (1997). Editing of glutamate receptor B subunit ion channel RNAs by four alternatively spliced DRADA2 double-stranded RNA adenosine deaminases. Mol. Cell. Biol..

[12] Gerber A., O’Connell M.A., Keller W. (1997). Two forms of human double-stranded RNA-specific editase 1 (hRED1) generated by the insertion of an Alu cassette. RNA.

[13] Melcher T., Maas S., Herb A., Sprengel R., Higuchi M., Seeburg P.H. (1996). RED2, a brain-specific member of the RNA-specific adenosine deaminase family. J. Biol. Chem..

[14] Gott J.M., Emeson R.B. (2000). Functions and mechanisms of RNA editing. Annu. Rev. Genet..

[15] Blum B., Bakalara N., Simpson L. (1990). A model for RNA editing in kinetoplastid mitochondria: “Guide” RNA molecules transcribed from maxicircle DNA provide the edited information. Cell.

[16] Cech T.R. (1991). RNA editing: World’s smallest introns?. Cell.

[17] Seiwert S.D., Heidmann S., Stuart K. (1996). Direct visualization of uridylate deletion in vitro suggests a mechanism for kinetoplastid RNA editing. Cell.

[18] Byrne E.M., Connell G.J., Simpson L. (1996). Guide RNA-directed uridine insertion RNA editing in vitro. EMBO J..

[19] Kable M.L., Seiwert S.D., Heidmann S., Stuart K. (1996). RNA editing: A mechanism for gRNA-specified uridylate insertion into precursor mRNA. Science.

[20] Ramaswami G., Li J.B. (2016). Identification of human RNA editing sites: A historical perspective. Methods.

[21] Lander E.S., Linton L.M., Birren B., Nusbaum C., Zody M.C., Baldwin J., Devon K., Dewar K., Doyle M., FitzHugh W. (2001). Initial sequencing and analysis of the human genome. Nature.

[22] Shendure J., Ji H. (2008). Next-generation DNA sequencing. Nat. Biotechnol..

[23] Bazak L., Haviv A., Barak M., Jacob-Hirsch J., Deng P., Zhang R., Isaacs F.J., Rechavi G., Li J.B., Eisenberg E. (2014). A-to-I RNA editing occurs at over a hundred million genomic sites, located in a majority of human genes. Genome Res..

[24] Andersen A.A., Panning B. (2003). Epigenetic gene regulation by noncoding RNAs. Curr. Opin. Cell Biol..

[25] Larriba E., del Mazo J. (2016). Role of Non-Coding RNAs in the Transgenerational Epigenetic Transmission of the Effects of Reprotoxicants. Int. J. Mol. Sci..

[26] Christofi T., Zaravinos A. (2019). RNA editing in the forefront of epitranscriptomics and human health. J. Transl. Med..

[27] Jain M., Jantsch M.F., Licht K. (2019). The Editor’s I on Disease Development. Trends Genet..

[28] Xu X., Wang Y., Liang H. (2018). The role of A-to-I RNA editing in cancer development. Curr. Opin. Genet. Dev..

[29] Kung C.P., Maggi L.B., Weber J.D. (2018). The Role of RNA Editing in Cancer Development and Metabolic Disorders. Front. Endocrinol. (Lausanne).

[30] Baysal B.E., Sharma S., Hashemikhabir S., Janga S.C. (2017). RNA Editing in Pathogenesis of Cancer. Cancer Res..

[31] Nishikura K. (2010). Functions and regulation of RNA editing by ADAR deaminases. Annu. Rev. Biochem..

[32] Nigita G., Marceca G.P., Tomasello L., Distefano R., Calore F., Veneziano D., Romano G., Nana-Sinkam S.P., Acunzo M., Croce C.M. (2019). ncRNA Editing: Functional Characterization and Computational Resources. Methods Mol. Biol..

[33] Matthews M.M., Thomas J.M., Zheng Y., Tran K., Phelps K.J., Scott A.I., Havel J., Fisher A.J., Beal P.A. (2016). Structures of human ADAR2 bound to dsRNA reveal base-flipping mechanism and basis for site selectivity. Nat. Struct. Mol. Biol..

[34] Goodman R.A., Macbeth M.R., Beal P.A. (2012). ADAR proteins: Structure and catalytic mechanism. Curr. Top. Microbiol. Immunol..

[35] Macbeth M.R., Schubert H.L., Vandemark A.P., Lingam A.T., Hill C.P., Bass B.L. (2005). Inositol hexakisphosphate is bound in the ADAR2 core and required for RNA editing. Science.

[36] Ryter J.M., Schultz S.C. (1998). Molecular basis of double-stranded RNA-protein interactions: Structure of a dsRNA-binding domain complexed with dsRNA. EMBO J..

[37] Stefl R., Xu M., Skrisovska L., Emeson R.B., Allain F.H. (2006). Structure and specific RNA binding of ADAR2 double-stranded RNA binding motifs. Structure.

[38] Chang K.Y., Ramos A. (2005). The double-stranded RNA-binding motif, a versatile macromolecular docking platform. FEBS J..

[39] Masliah G., Barraud P., Allain F.H. (2013). RNA recognition by double-stranded RNA binding domains: A matter of shape and sequence. Cell. Mol. Life Sci..

[40] Song C., Sakurai M., Shiromoto Y., Nishikura K. (2016). Functions of the RNA Editing Enzyme ADAR1 and Their Relevance to Human Diseases. Genes.

[41] Nie Y., Zhao Q., Su Y., Yang J.H. (2004). Subcellular distribution of ADAR1 isoforms is synergistically determined by three nuclear discrimination signals and a regulatory motif. J. Biol. Chem..

[42] Poulsen H., Nilsson J., Damgaard C.K., Egebjerg J., Kjems J. (2001). CRM1 mediates the export of ADAR1 through a nuclear export signal within the Z-DNA binding domain. Mol. Cell. Biol..

[43] Desterro J.M., Keegan L.P., Lafarga M., Berciano M.T., O’Connell M., Carmo-Fonseca M. (2003). Dynamic association of RNA-editing enzymes with the nucleolus. J. Cell Sci..

[44] Eckmann C.R., Neunteufl A., Pfaffstetter L., Jantsch M.F. (2001). The human but not the Xenopus RNA-editing enzyme ADAR1 has an atypical nuclear localization signal and displays the characteristics of a shuttling protein. Mol. Biol. Cell.

[45] Fritz J., Strehblow A., Taschner A., Schopoff S., Pasierbek P., Jantsch M.F. (2009). RNA-regulated interaction of transportin-1 and exportin-5 with the double-stranded RNA-binding domain regulates nucleocytoplasmic shuttling of ADAR1. Mol. Cell. Biol..

[46] Behm M., Wahlstedt H., Widmark A., Eriksson M., Ohman M. (2017). Accumulation of nuclear ADAR2 regulates adenosine-to-inosine RNA editing during neuronal development. J. Cell Sci..

[47] Consortium G.T. (2013). The Genotype-Tissue Expression (GTEx) project. Nat. Genet..

[48] Yang L., Huang P., Li F., Zhao L., Zhang Y., Li S., Gan Z., Lin A., Li W., Liu Y. (2012). c-Jun amino-terminal kinase-1 mediates glucose-responsive upregulation of the RNA editing enzyme ADAR2 in pancreatic beta-cells. PLoS ONE.

[49] Peng P.L., Zhong X., Tu W., Soundarapandian M.M., Molner P., Zhu D., Lau L., Liu S., Liu F., Lu Y. (2006). ADAR2-dependent RNA editing of AMPA receptor subunit GluR2 determines vulnerability of neurons in forebrain ischemia. Neuron.

[50] Tomaselli S., Galeano F., Alon S., Raho S., Galardi S., Polito V.A., Presutti C., Vincenti S., Eisenberg E., Locatelli F. (2015). Modulation of microRNA editing, expression and processing by ADAR2 deaminase in glioblastoma. Genome Biol..

[51] Chen C.X., Cho D.S., Wang Q., Lai F., Carter K.C., Nishikura K. (2000). A third member of the RNA-specific adenosine deaminase gene family, ADAR3, contains both single- and double-stranded RNA binding domains. RNA.

[52] Melcher T., Maas S., Herb A., Sprengel R., Seeburg P.H., Higuchi M. (1996). A mammalian RNA editing enzyme. Nature.

[53] Savva Y.A., Rieder L.E., Reenan R.A. (2012). The ADAR protein family. Genome Biol..

[54] Samuel C.E. (2011). Adenosine deaminases acting on RNA (ADARs) are both antiviral and proviral. Virology.

[55] Cho D.S., Yang W., Lee J.T., Shiekhattar R., Murray J.M., Nishikura K. (2003). Requirement of dimerization for RNA editing activity of adenosine deaminases acting on RNA. J. Biol. Chem..

[56] Tan M.H., Li Q., Shanmugam R., Piskol R., Kohler J., Young A.N., Liu K.I., Zhang R., Ramaswami G., Ariyoshi K. (2017). Dynamic landscape and regulation of RNA editing in mammals. Nature.

[57] Oakes E., Anderson A., Cohen-Gadol A., Hundley H.A. (2017). Adenosine Deaminase That Acts on RNA 3 (ADAR3) Binding to Glutamate Receptor Subunit B Pre-mRNA Inhibits RNA Editing in Glioblastoma. J. Biol. Chem..

[58] Paz-Yaacov N., Bazak L., Buchumenski I., Porath H.T., Danan-Gotthold M., Knisbacher B.A., Eisenberg E., Levanon E.Y. (2015). Elevated RNA Editing Activity Is a Major Contributor to Transcriptomic Diversity in Tumors. Cell Rep..

[59] Han L., Diao L., Yu S., Xu X., Li J., Zhang R., Yang Y., Werner H.M.J., Eterovic A.K., Yuan Y. (2015). The Genomic Landscape and Clinical Relevance of A-to-I RNA Editing in Human Cancers. Cancer Cell.

[60] Salter J.D., Bennett R.P., Smith H.C. (2016). The APOBEC Protein Family: United by Structure, Divergent in Function. Trends Biochem. Sci..

[61] Smith H.C. (2017). RNA binding to APOBEC deaminases; Not simply a substrate for C to U editing. RNA Biol..

[62] Salter J.D., Smith H.C. (2018). Modeling the Embrace of a Mutator: APOBEC Selection of Nucleic Acid Ligands. Trends Biochem. Sci..

[63] Smith H.C., Bennett R.P., Kizilyer A., McDougall W.M., Prohaska K.M. (2012). Functions and regulation of the APOBEC family of proteins. Semin. Cell Dev. Biol..

[64] Muramatsu M., Sankaranand V.S., Anant S., Sugai M., Kinoshita K., Davidson N.O., Honjo T. (1999). Specific expression of activation-induced cytidine deaminase (AID), a novel member of the RNA-editing deaminase family in germinal center B cells. J. Biol. Chem..

[65] Muramatsu M., Kinoshita K., Fagarasan S., Yamada S., Shinkai Y., Honjo T. (2000). Class switch recombination and hypermutation require activation-induced cytidine deaminase (AID), a potential RNA editing enzyme. Cell.

[66] Revy P., Muto T., Levy Y., Geissmann F., Plebani A., Sanal O., Catalan N., Forveille M., Dufourcq-Labelouse R., Gennery A. (2000). Activation-induced cytidine deaminase (AID) deficiency causes the autosomal recessive form of the Hyper-IgM syndrome (HIGM2). Cell.

[67] Navaratnam N., Sarwar R. (2006). An overview of cytidine deaminases. Int. J. Hematol..

[68] Arakawa H., Hauschild J., Buerstedde J.M. (2002). Requirement of the activation-induced deaminase (AID) gene for immunoglobulin gene conversion. Science.

[69] Greeve J., Altkemper I., Dieterich J.H., Greten H., Windler E. (1993). Apolipoprotein B mRNA editing in 12 different mammalian species: Hepatic expression is reflected in low concentrations of apoB-containing plasma lipoproteins. J. Lipid Res..

[70] Lerner T., Papavasiliou F.N., Pecori R. (2018). RNA Editors, Cofactors, and mRNA Targets: An Overview of the C-to-U RNA Editing Machinery and Its Implication in Human Disease. Genes.

[71] Chester A., Scott J., Anant S., Navaratnam N. (2000). RNA editing: Cytidine to uridine conversion in apolipoprotein B mRNA. Biochim. Biophys. Acta.

[72] Sato Y., Probst H.C., Tatsumi R., Ikeuchi Y., Neuberger M.S., Rada C. (2010). Deficiency in APOBEC2 leads to a shift in muscle fiber type, diminished body mass, and myopathy. J. Biol. Chem..

[73] Okuyama S., Marusawa H., Matsumoto T., Ueda Y., Matsumoto Y., Endo Y., Takai A., Chiba T. (2012). Excessive activity of apolipoprotein B mRNA editing enzyme catalytic polypeptide 2 (APOBEC2) contributes to liver and lung tumorigenesis. Int. J. Cancer.

[74] Chiu Y.L., Greene W.C. (2008). The APOBEC3 cytidine deaminases: An innate defensive network opposing exogenous retroviruses and endogenous retroelements. Annu. Rev. Immunol..

[75] Chen H., Lilley C.E., Yu Q., Lee D.V., Chou J., Narvaiza I., Landau N.R., Weitzman M.D. (2006). APOBEC3A is a potent inhibitor of adeno-associated virus and retrotransposons. Curr. Biol..

[76] Peng Z.G., Zhao Z.Y., Li Y.P., Wang Y.P., Hao L.H., Fan B., Li Y.H., Wang Y.M., Shan Y.Q., Han Y.X. (2011). Host apolipoprotein B messenger RNA-editing enzyme catalytic polypeptide-like 3G is an innate defensive factor and drug target against hepatitis C virus. Hepatology.

[77] Lada A.G., Krick C.F., Kozmin S.G., Mayorov V.I., Karpova T.S., Rogozin I.B., Pavlov Y.I. (2011). Mutator effects and mutation signatures of editing deaminases produced in bacteria and yeast. Biochemistry (Mosc.).

[78] Marino D., Perkovic M., Hain A., Jaguva Vasudevan A.A., Hofmann H., Hanschmann K.M., Muhlebach M.D., Schumann G.G., Konig R., Cichutek K. (2016). APOBEC4 Enhances the Replication of HIV-1. PLoS ONE.

[79] Yamanaka S., Balestra M.E., Ferrell L.D., Fan J., Arnold K.S., Taylor S., Taylor J.M., Innerarity T.L. (1995). Apolipoprotein B mRNA-editing protein induces hepatocellular carcinoma and dysplasia in transgenic animals. Proc. Natl. Acad. Sci. USA.

[80] Yamanaka S., Poksay K.S., Arnold K.S., Innerarity T.L. (1997). A novel translational repressor mRNA is edited extensively in livers containing tumors caused by the transgene expression of the apoB mRNA-editing enzyme. Genes Dev..

[81] Anant S., Davidson N.O. (2000). An AU-rich sequence element (UUUN[A/U]U) downstream of the edited C in apolipoprotein B mRNA is a high-affinity binding site for Apobec-1: Binding of Apobec-1 to this motif in the 3’ untranslated region of c-myc increases mRNA stability. Mol. Cell. Biol..

[82] Niavarani A., Shahrabi Farahani A., Sharafkhah M., Rassoulzadegan M. (2018). Pancancer analysis identifies prognostic high-APOBEC1 expression level implicated in cancer in-frame insertions and deletions. Carcinogenesis.

[83] Endo Y., Marusawa H., Kou T., Nakase H., Fujii S., Fujimori T., Kinoshita K., Honjo T., Chiba T. (2008). Activation-induced cytidine deaminase links between inflammation and the development of colitis-associated colorectal cancers. Gastroenterology.

[84] Matsumoto Y., Marusawa H., Kinoshita K., Endo Y., Kou T., Morisawa T., Azuma T., Okazaki I.M., Honjo T., Chiba T. (2007). Helicobacter pylori infection triggers aberrant expression of activation-induced cytidine deaminase in gastric epithelium. Nat. Med..

[85] Burns M.B., Lackey L., Carpenter M.A., Rathore A., Land A.M., Leonard B., Refsland E.W., Kotandeniya D., Tretyakova N., Nikas J.B. (2013). APOBEC3B is an enzymatic source of mutation in breast cancer. Nature.

[86] Burns M.B., Temiz N.A., Harris R.S. (2013). Evidence for APOBEC3B mutagenesis in multiple human cancers. Nat. Genet..

[87] Roberts S.A., Lawrence M.S., Klimczak L.J., Grimm S.A., Fargo D., Stojanov P., Kiezun A., Kryukov G.V., Carter S.L., Saksena G. (2013). An APOBEC cytidine deaminase mutagenesis pattern is widespread in human cancers. Nat. Genet..

[88] Cannataro V.L., Gaffney S.G., Sasaki T., Issaeva N., Grewal N.K.S., Grandis J.R., Yarbrough W.G., Burtness B., Anderson K.S., Townsend J.P. (2019). APOBEC-induced mutations and their cancer effect size in head and neck squamous cell carcinoma. Oncogene.

[89] Chan K., Roberts S.A., Klimczak L.J., Sterling J.F., Saini N., Malc E.P., Kim J., Kwiatkowski D.J., Fargo D.C., Mieczkowski P.A. (2015). An APOBEC3A hypermutation signature is distinguishable from the signature of background mutagenesis by APOBEC3B in human cancers. Nat. Genet..

[90] Liu H., Ma C.P., Chen Y.T., Schuyler S.C., Chang K.P., Tan B.C. (2014). Functional Impact of RNA editing and ADARs on regulation of gene expression: Perspectives from deep sequencing studies. Cell Biosci..

[91] Rahnamoun H., Orozco P., Lauberth S.M. (2020). The role of enhancer RNAs in epigenetic regulation of gene expression. Transcription.

[92] Hombach S., Kretz M. (2016). Non-coding RNAs: Classification, Biology and Functioning. Adv. Exp. Med. Biol..

[93] Fu X.D. (2014). Non-coding RNA: A new frontier in regulatory biology. Natl. Sci. Rev..

[94] Romano G., Veneziano D., Acunzo M., Croce C.M. (2017). Small non-coding RNA and cancer. Carcinogenesis.

[95] Nishikura K. (2016). A-to-I editing of coding and non-coding RNAs by ADARs. Nat. Rev. Mol. Cell. Biol..

[96] Cannell I.G., Kong Y.W., Bushell M. (2008). How do microRNAs regulate gene expression?. Biochem. Soc. Trans..

[97] Bartel D.P. (2004). MicroRNAs: Genomics, biogenesis, mechanism, and function. Cell.

[98] Yang W., Chendrimada T.P., Wang Q., Higuchi M., Seeburg P.H., Shiekhattar R., Nishikura K. (2006). Modulation of microRNA processing and expression through RNA editing by ADAR deaminases. Nat. Struct. Mol. Biol..

[99] Kawahara Y., Zinshteyn B., Chendrimada T.P., Shiekhattar R., Nishikura K. (2007). RNA editing of the microRNA-151 precursor blocks cleavage by the Dicer-TRBP complex. EMBO Rep..

[100] Kawahara Y., Megraw M., Kreider E., Iizasa H., Valente L., Hatzigeorgiou A.G., Nishikura K. (2008). Frequency and fate of microRNA editing in human brain. Nucleic Acids Res..

[101] Kozomara A., Birgaoanu M., Griffiths-Jones S. (2019). miRBase: From microRNA sequences to function. Nucleic Acids Res..

[102] Acunzo M., Romano G., Wernicke D., Croce C.M. (2015). MicroRNA and cancer--a brief overview. Adv. Biol. Regul..

[103] Nigita G., Acunzo M., Romano G., Veneziano D., Lagana A., Vitiello M., Wernicke D., Ferro A., Croce C.M. (2016). microRNA editing in seed region aligns with cellular changes in hypoxic conditions. Nucleic Acids Res..

[104] Kawahara Y., Zinshteyn B., Sethupathy P., Iizasa H., Hatzigeorgiou A.G., Nishikura K. (2007). Redirection of silencing targets by adenosine-to-inosine editing of miRNAs. Science.

[105] Van der Kwast R., van Ingen E., Parma L., Peters H.A.B., Quax P.H.A., Nossent A.Y. (2018). Adenosine-to-Inosine Editing of MicroRNA-487b Alters Target Gene Selection After Ischemia and Promotes Neovascularization. Circ. Res..

[106] Pinto Y., Buchumenski I., Levanon E.Y., Eisenberg E. (2018). Human cancer tissues exhibit reduced A-to-I editing of miRNAs coupled with elevated editing of their targets. Nucleic Acids Res..

[107] Wang Y., Xu X., Yu S., Jeong K.J., Zhou Z., Han L., Tsang Y.H., Li J., Chen H., Mangala L.S. (2017). Systematic characterization of A-to-I RNA editing hotspots in microRNAs across human cancers. Genome Res..

[108] Nigita G., Distefano R., Veneziano D., Romano G., Rahman M., Wang K., Pass H., Croce C.M., Acunzo M., Nana-Sinkam P. (2018). Tissue and exosomal miRNA editing in Non-Small Cell Lung Cancer. Sci. Rep..

[109] Galore-Haskel G., Nemlich Y., Greenberg E., Ashkenazi S., Hakim M., Itzhaki O., Shoshani N., Shapira-Fromer R., Ben-Ami E., Ofek E. (2015). A novel immune resistance mechanism of melanoma cells controlled by the ADAR1 enzyme. Oncotarget.

[110] Liu W.H., Chen C.H., Yeh K.H., Li C.L., Wu Y.J., Chen D.S., Chen P.J., Yeh S.H. (2013). ADAR2-mediated editing of miR-214 and miR-122 precursor and antisense RNA transcripts in liver cancers. PLoS ONE.

[111] Cesarini V., Silvestris D.A., Tassinari V., Tomaselli S., Alon S., Eisenberg E., Locatelli F., Gallo A. (2018). ADAR2/miR-589-3p axis controls glioblastoma cell migration/invasion. Nucleic Acids Res..

[112] Choudhury Y., Tay F.C., Lam D.H., Sandanaraj E., Tang C., Ang B.T., Wang S. (2012). Attenuated adenosine-to-inosine editing of microRNA-376a* promotes invasiveness of glioblastoma cells. J. Clin. Investig..

[113] Allegra D., Bilan V., Garding A., Dohner H., Stilgenbauer S., Kuchenbauer F., Mertens D., Zucknick M. (2014). Defective DROSHA processing contributes to downregulation of MiR-15/-16 in chronic lymphocytic leukemia. Leukemia.

[114] Luciano D.J., Mirsky H., Vendetti N.J., Maas S. (2004). RNA editing of a miRNA precursor. RNA.

[115] Blow M.J., Grocock R.J., van Dongen S., Enright A.J., Dicks E., Futreal P.A., Wooster R., Stratton M.R. (2006). RNA editing of human microRNAs. Genome Biol..

[116] Nigita G., Veneziano D., Ferro A. (2015). A-to-I RNA Editing: Current Knowledge Sources and Computational Approaches with Special Emphasis on Non-Coding RNA Molecules. Front. Bioeng. Biotechnol..

[117] Distefano R., Nigita G., Veneziano D., Romano G., Croce C.M., Acunzo M. (2019). isoTar: Consensus Target Prediction with Enrichment Analysis for MicroRNAs Harboring Editing Sites and Other Variations. Methods Mol. Biol..

[118] Kiran A.M., O’Mahony J.J., Sanjeev K., Baranov P.V. (2013). Darned in 2013: Inclusion of model organisms and linking with Wikipedia. Nucleic Acids Res..

[119] Ramaswami G., Li J.B. (2014). RADAR: A rigorously annotated database of A-to-I RNA editing. Nucleic Acids Res..

[120] Alon S., Mor E., Vigneault F., Church G.M., Locatelli F., Galeano F., Gallo A., Shomron N., Eisenberg E. (2012). Systematic identification of edited microRNAs in the human brain. Genome Res..

[121] Alon S., Eisenberg E. (2013). Identifying RNA editing sites in miRNAs by deep sequencing. Methods Mol. Biol..

[122] Alon S., Erew M., Eisenberg E. (2015). DREAM: A webserver for the identification of editing sites in mature miRNAs using deep sequencing data. Bioinformatics.

[123] Lu Y., Baras A.S., Halushka M.K. (2018). miRge 2.0 for comprehensive analysis of microRNA sequencing data. BMC Bioinform..

[124] Kiran A., Baranov P.V. (2010). DARNED: A DAtabase of RNa EDiting in humans. Bioinformatics.

[125] Picardi E., D’Erchia A.M., Lo Giudice C., Pesole G. (2017). REDIportal: A comprehensive database of A-to-I RNA editing events in humans. Nucleic Acids Res..

[126] Ma L., Bajic V.B., Zhang Z. (2013). On the classification of long non-coding RNAs. RNA Biol..

[127] Volders P.J., Anckaert J., Verheggen K., Nuytens J., Martens L., Mestdagh P., Vandesompele J. (2019). LNCipedia 5: Towards a reference set of human long non-coding RNAs. Nucleic Acids Res..

[128] Quek X.C., Thomson D.W., Maag J.L., Bartonicek N., Signal B., Clark M.B., Gloss B.S., Dinger M.E. (2015). lncRNAdb v2.0: Expanding the reference database for functional long noncoding RNAs. Nucleic Acids Res..

[129] Mongelli A., Martelli F., Farsetti A., Gaetano C. (2019). The Dark That Matters: Long Non-coding RNAs as Master Regulators of Cellular Metabolism in Non-communicable Diseases. Front. Physiol..

[130] Lukiw W.J., Handley P., Wong L., Crapper McLachlan D.R. (1992). BC200 RNA in normal human neocortex, non-Alzheimer dementia (NAD), and senile dementia of the Alzheimer type (AD). Neurochem. Res..

[131] Ishii N., Ozaki K., Sato H., Mizuno H., Susumu S., Takahashi A., Miyamoto Y., Ikegawa S., Kamatani N., Hori M. (2006). Identification of a novel non-coding RNA, MIAT, that confers risk of myocardial infarction. J. Hum. Genet..

[132] Gupta R.A., Shah N., Wang K.C., Kim J., Horlings H.M., Wong D.J., Tsai M.C., Hung T., Argani P., Rinn J.L. (2010). Long non-coding RNA HOTAIR reprograms chromatin state to promote cancer metastasis. Nature.

[133] Yang Y., Zhou X., Jin Y. (2013). ADAR-mediated RNA editing in non-coding RNA sequences. Sci. China Life Sci..

[134] Prasanth K.V., Prasanth S.G., Xuan Z., Hearn S., Freier S.M., Bennett C.F., Zhang M.Q., Spector D.L. (2005). Regulating gene expression through RNA nuclear retention. Cell.

[135] Peng Z., Cheng Y., Tan B.C., Kang L., Tian Z., Zhu Y., Zhang W., Liang Y., Hu X., Tan X. (2012). Comprehensive analysis of RNA-Seq data reveals extensive RNA editing in a human transcriptome. Nat. Biotechnol..

[136] Salameh A., Lee A.K., Cardo-Vila M., Nunes D.N., Efstathiou E., Staquicini F.I., Dobroff A.S., Marchio S., Navone N.M., Hosoya H. (2015). PRUNE2 is a human prostate cancer suppressor regulated by the intronic long noncoding RNA PCA3. Proc. Natl. Acad. Sci. USA.

[137] Luo H., Fang S., Sun L., Liu Z., Zhao Y. (2017). Comprehensive Characterization of the RNA Editomes in Cancer Development and Progression. Front. Genet..

[138] Su A.A., Randau L. (2011). A-to-I and C-to-U editing within transfer RNAs. Biochemistry (Mosc.).

[139] Torres A.G., Pineyro D., Filonava L., Stracker T.H., Batlle E., Ribas de Pouplana L. (2014). A-to-I editing on tRNAs: Biochemical, biological and evolutionary implications. FEBS Lett..

[140] Asaoka M., Ishikawa T., Takabe K., Patnaik S.K. (2019). APOBEC3-Mediated RNA Editing in Breast Cancer is Associated with Heightened Immune Activity and Improved Survival. Int. J. Mol. Sci..

[141] Zhang M., Fritsche J., Roszik J., Williams L.J., Peng X., Chiu Y., Tsou C.C., Hoffgaard F., Goldfinger V., Schoor O. (2018). RNA editing derived epitopes function as cancer antigens to elicit immune responses. Nat. Commun..

[142] Samuel C.E. (2019). Adenosine deaminase acting on RNA (ADAR1), a suppressor of double-stranded RNA-triggered innate immune responses. J. Biol. Chem..

[143] Anadon C., Guil S., Simo-Riudalbas L., Moutinho C., Setien F., Martinez-Cardus A., Moran S., Villanueva A., Calaf M., Vidal A. (2016). Gene amplification-associated overexpression of the RNA editing enzyme ADAR1 enhances human lung tumorigenesis. Oncogene.

[144] Chan T.H., Qamra A., Tan K.T., Guo J., Yang H., Qi L., Lin J.S., Ng V.H., Song Y., Hong H. (2016). ADAR-Mediated RNA Editing Predicts Progression and Prognosis of Gastric Cancer. Gastroenterology.

[145] Qin Y.R., Qiao J.J., Chan T.H., Zhu Y.H., Li F.F., Liu H., Fei J., Li Y., Guan X.Y., Chen L. (2014). Adenosine-to-inosine RNA editing mediated by ADARs in esophageal squamous cell carcinoma. Cancer Res..

[146] Jiang Q., Crews L.A., Barrett C.L., Chun H.J., Court A.C., Isquith J.M., Zipeto M.A., Goff D.J., Minden M., Sadarangani A. (2013). ADAR1 promotes malignant progenitor reprogramming in chronic myeloid leukemia. Proc. Natl. Acad. Sci. USA.

[147] Zipeto M.A., Court A.C., Sadarangani A., Delos Santos N.P., Balaian L., Chun H.J., Pineda G., Morris S.R., Mason C.N., Geron I. (2016). ADAR1 Activation Drives Leukemia Stem Cell Self-Renewal by Impairing Let-7 Biogenesis. Cell Stem Cell.

[148] Manguso R.T., Pope H.W., Zimmer M.D., Brown F.D., Yates K.B., Miller B.C., Collins N.B., Bi K., LaFleur M.W., Juneja V.R. (2017). In vivo CRISPR screening identifies Ptpn2 as a cancer immunotherapy target. Nature.

[149] Ishizuka J.J., Manguso R.T., Cheruiyot C.K., Bi K., Panda A., Iracheta-Vellve A., Miller B.C., Du P.P., Yates K.B., Dubrot J. (2019). Loss of ADAR1 in tumours overcomes resistance to immune checkpoint blockade. Nature.

[150] Caponio V.C.A., Troiano G., Botti G., Pedicillo M.C., Lo Russo L., Mastrangelo F., Ciavarella D., Losito N.S., Aquino G., Nocini R. (2019). Overexpression of ADAR1 into the cytoplasm correlates with a better prognosis of patients with oral squamous cells carcinoma. J. Oral Pathol. Med..

[151] Lee S.H., Kim H.P., Kang J.K., Song S.H., Han S.W., Kim T.Y. (2017). Identification of Diverse Adenosine-to-Inosine RNA Editing Subtypes in Colorectal Cancer. Cancer Res. Treat..

[152] Permuth J.B., Reid B., Earp M., Chen Y.A., Monteiro A.N., Chen Z., Group A.S., Chenevix-Trench G., Fasching P.A., Beckmann M.W. (2016). Inherited variants affecting RNA editing may contribute to ovarian cancer susceptibility: Results from a large-scale collaboration. Oncotarget.

[153] Mo F., Wyatt A.W., Sun Y., Brahmbhatt S., McConeghy B.J., Wu C., Wang Y., Gleave M.E., Volik S.V., Collins C.C. (2014). Systematic identification and characterization of RNA editing in prostate tumors. PLoS ONE.

[154] Saiselet M., Gacquer D., Spinette A., Craciun L., Decaussin-Petrucci M., Andry G., Detours V., Maenhaut C. (2015). New global analysis of the microRNA transcriptome of primary tumors and lymph node metastases of papillary thyroid cancer. BMC Genom..

[155] Hezaveh K., Kloetgen A., Bernhart S.H., Mahapatra K.D., Lenze D., Richter J., Haake A., Bergmann A.K., Brors B., Burkhardt B. (2016). Alterations of microRNA and microRNA-regulated messenger RNA expression in germinal center B-cell lymphomas determined by integrative sequencing analysis. Haematologica.

[156] Maemura K., Watanabe K., Ando T., Hiyama N., Sakatani T., Amano Y., Kage H., Nakajima J., Yatomi Y., Nagase T. (2018). Altered editing level of microRNAs is a potential biomarker in lung adenocarcinoma. Cancer Sci..

[157] Velazquez-Torres G., Shoshan E., Ivan C., Huang L., Fuentes-Mattei E., Paret H., Kim S.J., Rodriguez-Aguayo C., Xie V., Brooks D. (2018). A-to-I miR-378a-3p editing can prevent melanoma progression via regulation of PARVA expression. Nat. Commun..

